# Predictive and discriminative localization of pathology using high resolution class activation maps with CNNs

**DOI:** 10.7717/peerj-cs.622

**Published:** 2021-07-14

**Authors:** Sumeet Shinde, Priyanka Tupe-Waghmare, Tanay Chougule, Jitender Saini, Madhura Ingalhalikar

**Affiliations:** 1 Symbiosis Center for Medical Image Analysis, Symbiosis International (Deemed University), Pune, Maharashtra, India; 2 Symbiosis Institute of Technology, Symbiosis International (Deemed University), Pune, Maharashtra, India; 3 Department of Radiology, National Institute of Mental Health and Neurosciences, Bangalore, India

**Keywords:** Class activation maps, Discriminative, Localization, High resolution, MURA, ISIC, Parkinson’s disease

## Abstract

**Purpose:**

Existing class activation mapping (CAM) techniques extract the feature maps only from a single layer of the convolutional neural net (CNN), generally from the final layer and then interpolate to upsample to the original image resolution to locate the discriminative regions. Consequently these provide a coarse localization that may not be able to capture subtle abnormalities in medical images. To alleviate this, our work proposes a technique called high resolution class activation mapping (HR-CAMs) that can provide enhanced visual explainability to the CNN models.

**Methods:**

HR-CAMs fuse feature maps by training a network using the input from multiple layers of a trained CNN, thus gaining information from every layer that can localize abnormalities with greater details in original image resolution. The technique is validated qualitatively and quantitatively on a simulated dataset of 8,000 images followed by applications on multiple image analysis tasks that include (1) skin lesion classification (ISIC open dataset—25,331 cases) and (2) predicting bone fractures (MURA open dataset—40,561 images) (3) predicting Parkinson’s disease (PD) from neuromelanin sensitive MRI (small cohort-80 subjects).

**Results:**

We demonstrate that our model creates clinically interpretable subject specific high resolution discriminative localizations when compared to widely used CAMs and Gradient-CAMs.

**Conclusion:**

HR-CAMs provide finer delineation of abnormalities thus facilitating superior explainability to CNNs as has been demonstrated from its rigorous validation.

## Introduction

Convolutional neural nets (CNNs) have demonstrated a superior performance on image based predictive tasks, particularly on large sample sizes when compared to statistical learning algorithms applied on hand-picked or empirically drawn features from the images (Krizhevsky, Sutskever & Hinton, 2017; Goodfellow et al., 2016). However, there is a lack of explanation in terms of the CNN learning process to identify precise regions of the image that are being used in the discriminative process. This is crucial in medical imaging, where the regions of interest facilitate clinical interpretability and have the potential to support prognosis, risk prediction and improve treatment efficacy.

Discriminative localization on images was earlier based on mapping the CNN activations back to the input space using the technique of deconvolution (Zeiler & Fergus, 2014), by masking out regions to extract the regions with maximal activations (Bazzani et al., 2016) or by visual encoding of CNNs by inverting the deep features (Mahendran & Vedaldi, 2015). However, current methods rely on class activation maps (CAM) that are more straightforward and exhibit capability to highlight the discriminative regions by either using global average pooling (Zhou et al., 2016) after the final convolutional layer or using gradient information (Grad-CAM) from any intermediate or final layer (Selvaraju et al., 2017) and their variants (Chattopadhay et al., 2018; Zhao et al., 2018).

The most prevailing issue with using CAMs is that they extract information only from a single layer of the CNN. Majority applications have employed the final layer as it entails the most abstract markers and can therefore facilitate more accurate maps than using any intermediate layer. However, especially in medical imaging where the original images itself are not super high-resolution, the final layer is usually a highly down sampled layer and therefore provides a low resolution CAM which when interpolated to the original image size results into a coarse map that fails to capture subtle and diffuse patterns of abnormalities that delineate the classes under consideration. To overcome these challenges, we propose a novel technique called HR-CAM to visualize and underscore the discriminative regions with high precision. We achieve this by capturing feature maps from multiple layers (pre-max-pooling layers) and aggregating these for a classifier. HR-CAMs have the ability to identify precise regions of delineation and can be employed via any CNN architecture. Unlike CAMs proposed by Zhou et al. (2016) which capture only the high level information from the activations of the last convolutional layer, HR-CAMs incorporate low level, intermediate as well as high level of information from the CNN layers corresponding to the depth of the model. This work employs HR-CAMs on a large-scale simulated data, open benchmark datasets of ISIC and MURA as well as on a small sample-size of neuromelanin sensitive MRI for PD. We apply multiple models to these datasets and demonstrate that the technique provides precise maps regardless of the application, training model and sample size under consideration.

## Methods

### HR-CAMs

**Figure 1:**
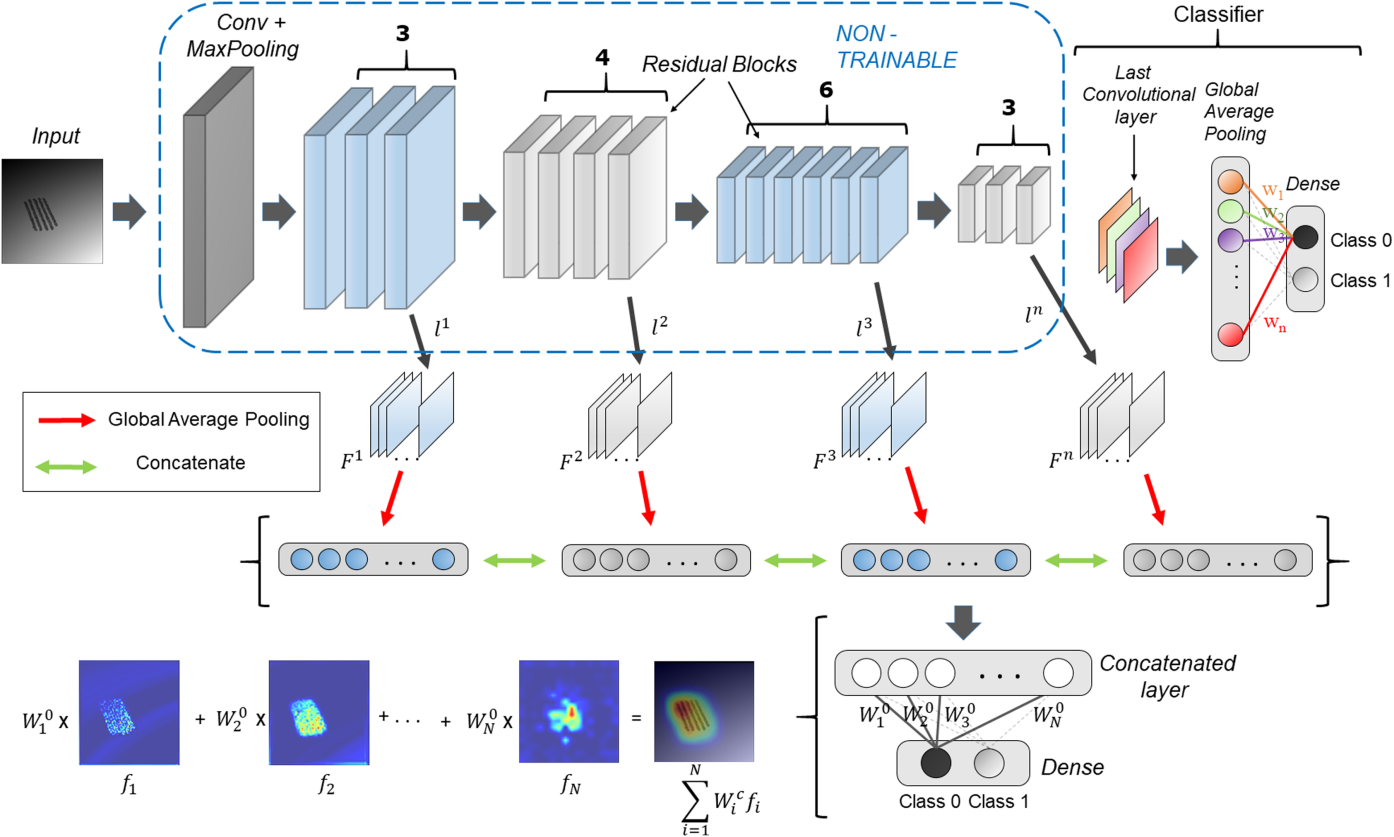
HR-CAM architecture using ResNet-50 as the base model. The convolutional layers are set to non-trainable after initially training the ResNet-50 based classifier. Intermediate layers are sampled and their average pooled values are stacked together to form one single vector for optimization.

Figure 1 shows the schematic overview of the HR-CAM architecture. The base CNN architecture is first trained using the train-data with a global average pool (GAP) layer following the final convolutional layer. This GAP layer is followed by a ‘softmax’ based layer and generates normalized prediction scores pertaining to every class. This method is generalizable to any CNN architecture irrespective of the model depth and number of training parameters.

The generation of HR-CAMs requires training the modified CNN architecture by setting the convolutional layers to non-trainable as described below. Arbitrarily, we choose *k* number of convolutional layers *L = {*}{}{l^1},}{}{l^2}*,…*,}{}{l^k}*}*, such that the set *L* consists of convolutional layers which constitute the low-level, intermediate and high level of encoded information from the images. Let the feature maps corresponding to these layers be }{}{F^i} *= {*}{}f_1^i,}{}f_2^i*…*}{},f_m^i*}*. The feature maps are outputs of various convolutional kernels applied on the feature maps of the preceding layers. The number of feature maps corresponding to the *k*^th^ layer is given by }{}{m_k}. These feature maps are two-dimensional spatial representations of the encoded information and are fed as input to a global average pooling layer. The GAP layer maps them to a single value based on the activation values globally. These average pooled values corresponding to feature maps from all the selected convolutional layers are stacked together as shown in Fig. 1 to form a concatenated vector of length *N* where *N* is given by Eq. (1).

(1) }{}N = \; \mathop \sum \limits_{i = 1}^k {m_i}

Following the concatenated layer is a single fully-connected layer which is trained to minimize the categorical cross entropy loss function (Eq. (2)) where }{}{y_n} is target output probability, }{}{\hat y_n} is predicted output probability, *S* is number of samples and }{}J\left( w \right) is categorical cross-entropy loss and is achieved by using the Adam optimizer. The optimization is performed by freezing the convolutional layers.

(2) }{}J\left( w \right) = \displaystyle{{ - 1} \over S}\mathop \sum \limits_{n = 1}^S [{y_n}\log {\hat y_n} + \; \left( {1 - {y_n}} \right)\log \left( {1 - {{\hat y}_n}} \right)]

This assigns new weights, }{}{W^c} *= {*}{}w_1^c,}{}w_2^c*…*}{},w_n^c*}* to all the values in the concatenated layer and ultimately the corresponding feature maps according to their importance for classification. These resulting weights pertaining to every class, support weighted aggregation of the feature maps.

Finally, to obtain the discriminative activations, we forward propagate the input image to acquire the intermediate convolutional outputs (}{}{l^1},}{}{l^2}*,…*,}{}{l^k}) at every chosen convolutional layer and weights (}{}w_1^c,}{}w_2^c*…*}{},w_n^c) at the output layer for the respective class, as given in Zhou et al. (2016). The resulting convolutional outputs consist of feature maps of varying spatial resolutions. Bilinear interpolation is used to upsample these feature maps to match the input image resolution. To create the class activation map, the weights \; w_i^c\; for the respective class are multiplied with the feature maps }{}{f_i} and then added together, as shown in Fig. 1. The resulting class activation map *A* can therefore demonstrate the most discerning regions in the image in high resolution.

(3) }{}A = \; \mathop \sum \limits_{i = 1}^N W_i^c{f_i}

Although Fig. 1 uses Resnet50 architecture with global average pooling (GAP) and fully connected layers for binary classification, the proposed technique is generalizable to any CNN based classifier.

### Datasets

The following sections describe the datasets used in this work.

#### Simulated data

Simulated dataset consisted of two classes of images. Class 1 comprised of images with normative data with added random noise whereas the second class consisted of images with simulated pathology in addition to random noise. These abnormalities in latter class were localized as well as diffuse in nature. Both classes contained 4,000 images each. Examples of these images are illustrated in Fig. 1.

#### ISIC

This dataset consisted of images from the ISIC archive (https://www.isic-archive.com/), an international repository of dermoscopic images developed by the International Skin Imaging Collaboration (ISIC) which includes images belonging to eight classes of skin lesions as follows: Melanoma, Melanocytic nevus, Basal cell carcinoma, Actinic keratosis, Benign keratosis, Dermatofibroma, Vascular lesion, Squamous cell carcinoma. The ISIC archive is a collection of multiple databases and currently includes a total of 25,331 images from BCN_20000 (Combalia et al., 2019), HAM10000 (Tschandl, Rosendahl & Kittler, 2018) and MSK (Codella et al., 2019) datasets. For the BCN_20000, images were obtained from the Department of Dermatology at the “Hospital Clínic de Barcelona” using a set of dermoscopic attachments on three high-resolution cameras that were stored using a directory structure in a server of the hospital. Images from the HAM10000 dataset were obtained from the Department of Dermatology, Vienna, Austria with a MoleMax HD system which had a resolution of 1,872 × 1,053 px with non-quadratic pixels. Finally, around 2,000 images of skin lesions were added from the ISIC 2017: Skin Lesion Analysis towards Melanoma Detection challenge (Marchetti et al., 2020).

#### Neuromelanin MRI for PD

The PD dataset included neuromelanin sensitive MRI (NMS-MRI) scanned on Philips 3T Inginea^TM^ with a 32-channel head coil. The protocol involved a fast spin echo T1 sequence using TR/TE: 26/2.2 ms, flip angle: 20°, reconstructed matrix size: 512 × 512; field of view: 180 × 180 × 50mm, for 45 patients with Parkinson’s disease (PD) and 35 age and gender matched controls. The NMS-MRI provides a good contrast in the substantia nigra pars compacta (SNc) and is useful in identifying PD as PD manifests depigmentation of the SNc (Sasaki et al., 2006; Takahashi et al., 2018).

#### MURA

MURA (**mu**sculoskeletal **ra**diographs) is one of the largest public radiographic image datasets collected from HIPAA-compliant images from the Picture Archive and Communication System (PACS) of Stanford Hospital (https://stanfordmlgroup.github.io/competitions/mura/) (Rajpurkar et al., 2017). It consists of 14,863 studies from 12,173 patients, with a total of 40,561 multi-view radiographic images. Each belongs to one of seven standard upper extremity radiographic study types: elbow, finger, forearm, hand, humerus, shoulder and wrist, which are manually labeled by radiologists as either normal or abnormal (Rajpurkar et al., 2017). The aim of this dataset is to label the image with bone fracture and localize the fracture on the image.

### Comparative methods

HR-CAMs were compared with the current state-of-art techniques: (1) GAP based CAMs by Zhou et al. (2016) and (2) Grad-CAMs (Selvaraju et al., 2017). The first method by Zhou et al. (2016) uses the feature maps from the final convolutional layer to obtain the CAMs. These feature maps are then average pooled globally to obtain a vector for optimization. The CAM is obtained by the weighted aggregation of these feature maps. This method necessarily uses feature maps only from the last convolutional layer.

Gradient-weighted Class Activation Mapping (Grad-CAM), uses the gradients of the target class, flowing into any convolutional layer to produce a coarse localization map highlighting the important regions in the image for predicting that particular class. In our case we use a convolutional layer from the pre-final convolutional block to implement Grad-CAM (which has been generally applied). Any of the single intermediate convolutional layers can also be used in the implementation of Grad-CAM.

## Results

Results for each of the datasets are provided in the following subsections.

### Simulated data

**Figure 2:**
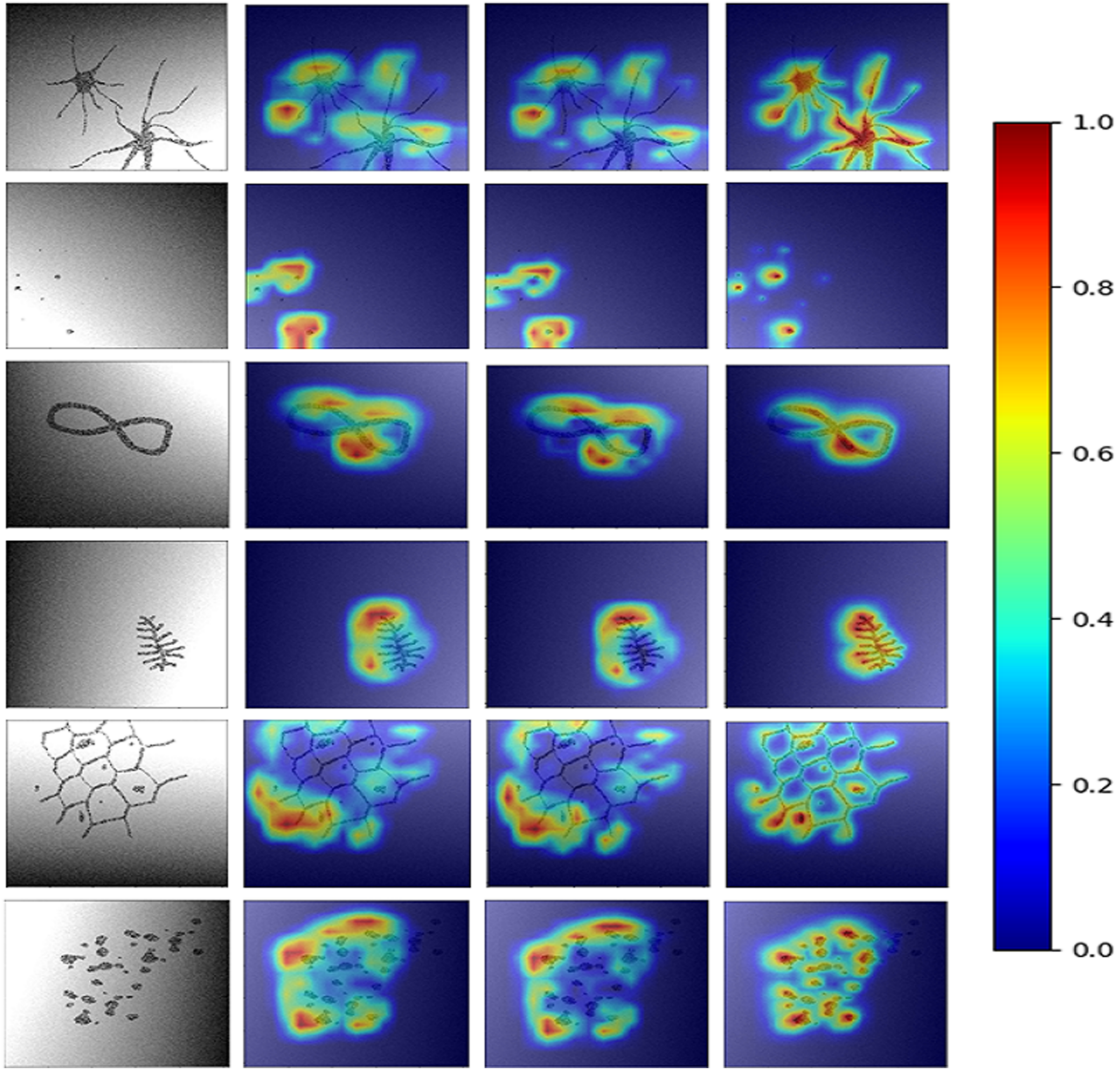
Results for all the three CAM techniques. First column shows the input image. CAM’s obtained by Zhou et al.’s (2016) method are shown in column 2. The results for Grad-CAMs and HR-CAMs are shown in the subsequent columns.

To obtain HR-CAMs, ResNet-50 architecture was employed as the base model. Data augmentation techniques involving shift, flip and rotation of images were used to increase the simulated dataset size and make the model robust. This setup was trained on 6,000 images and validated on 2,000 distinct simulated images to obtain a validation accuracy of 95% (AU-ROC = 0.99) using binary cross-entropy as the loss metric. The results for the CAMs are shown in Fig. 2. Qualitatively, it can be seen that HR-CAMs could delineate the distinguishing components/abnormalities with greater precision compared to Zhou’s CAMs and Grad-CAM which provide coarse localization of the abnormality.

**Figure 3:**
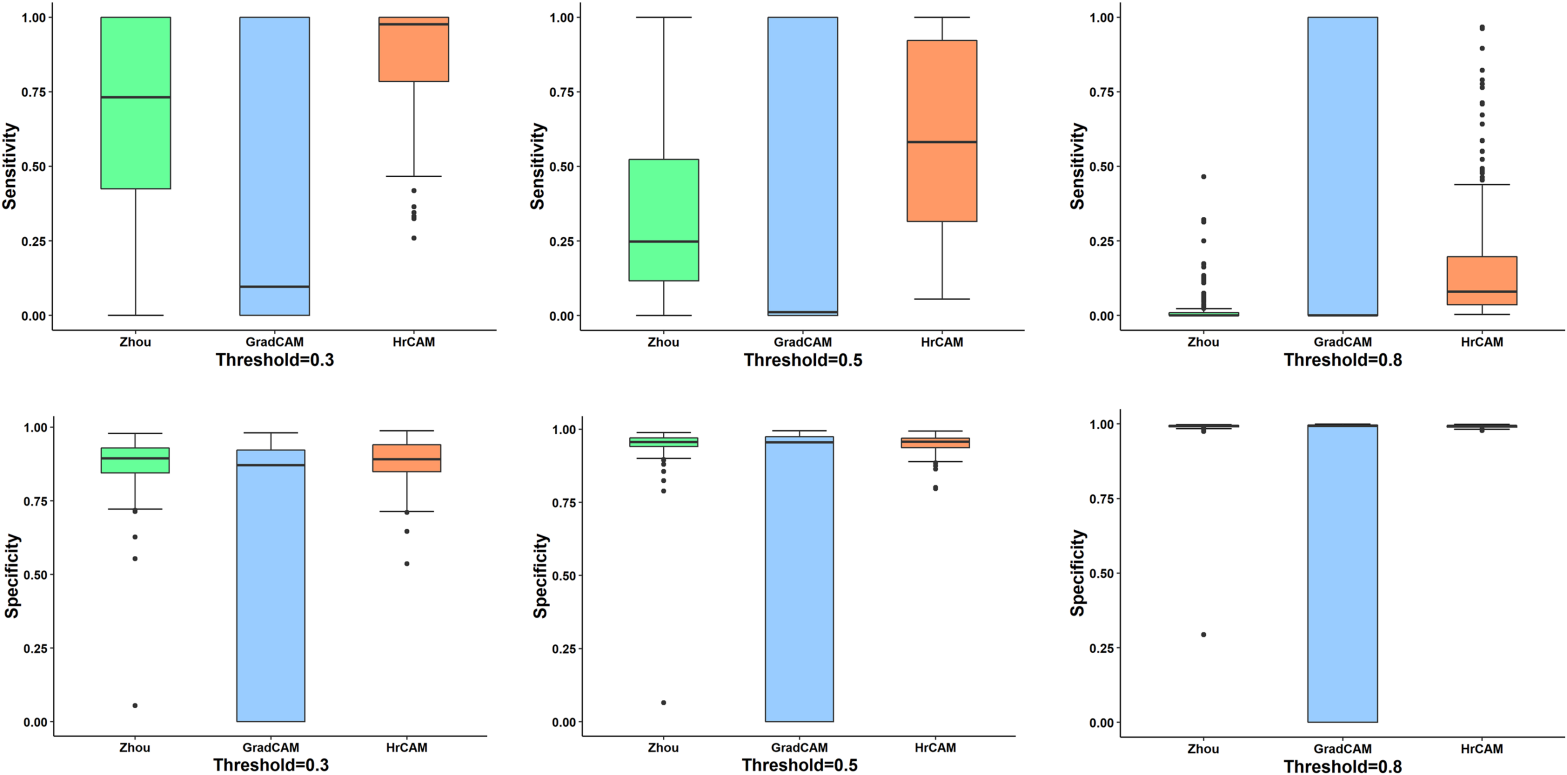
The sensitivity and specificity plots for all the three CAM techniques—Zhou’s CAM, HR-CAMs, GradCAM over varying intensity thresholds are shown. Median values for sensitivity and specificity are higher for HR-CAMs.

Figure 3 illustrates the quantitative comparison between the three methods based on a ground truth mask that was generated along with all simulated abnormalities for all the testing images. CAMs for the testing data were normalized (min-max normalization) between 0 to 1. Varying the intensity thresholds for these CAMs, the pixel wise sensitivity (true positive rate) and specificity (true negative rate) scores were computed and plotted at three different thresholds: 0.3, 0.5 and 0.8. It can be observed that the median values for the sensitivity and specificity were higher for HR-CAMs in comparison to the other two methods. A test for significance (t-test) was performed between HR-CAMs vs. Zhou’s CAMs and HR-CAMs vs. GradCAM which demonstrated that the sensitivity (*p*-value < 0.0001 and *p*-value < 0.0001 respectively) was significantly different however specificity between HR-CAM and Zhou’s CAM did not vary (*p*-value > 0.05) conversely between HR-CAM and GradCAM it was highly significant (*p*-value < 0.0001).

### ISIC

The ISIC dataset posed a multi-class classification problem. Since the data belonged to 8 different classes, categorical cross-entropy was used as the loss metric for two separate models under consideration. Model 1 used transfer learning on the ResNet-50 (as shown in Fig. 1) base model architecture by initializing it with weights from ‘IMAGENET’ (Krizhevsky, Sutskever & Hinton, 2017) along with augmentation techniques which included flipping, shifting and rotation of images while Model 2 used a basic VGG-19 architecture that is characterized by its simplicity, using only 3 × 3 convolutional layers stacked on top of each other in increasing depth (Simonyan & Zisserman, 2014). We initialized the model using ‘IMAGENET’ weights and augmentation techniques as described for model 1.

**Figure 4:**
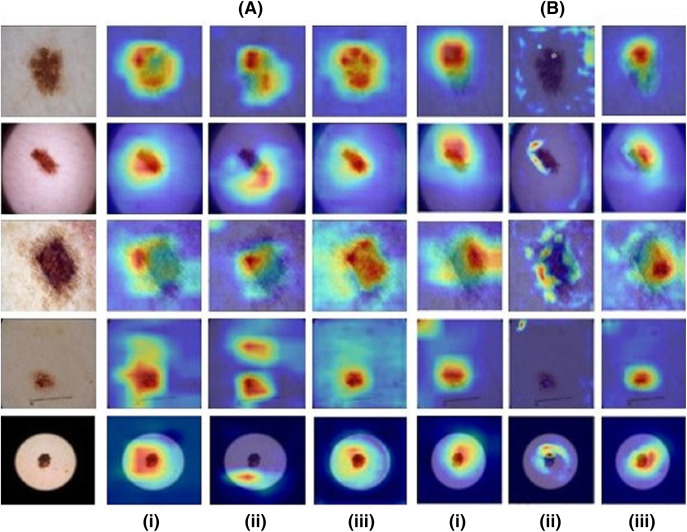
Results for images from the ISIC dataset compared across all the CAM methods. First column shows the input image. (A) depicts the ResNet-50 base model and (B) depicts the VGG-19 base model for (i) the Zhou method, (ii) Grad CAM and (iii) HR-CAM.

Model 1 resulted in an accuracy of 75.35% and a micro-averaged F1-score of 0.75 for the base model while model 2 performed with an 77.95% accuracy and 0.73 F1-score. However, as illustrated by Figs. 4A and 4B  the CAMs for Zhou and Grad-CAMs were unable to focus precisely on the lesional patch and resulted in a coarse blob on and around the lesion, whereas HR-CAMs were able to capture the exact patches of the skin lesions.

### Neuromelanin MRI for PD

**Figure 5:**
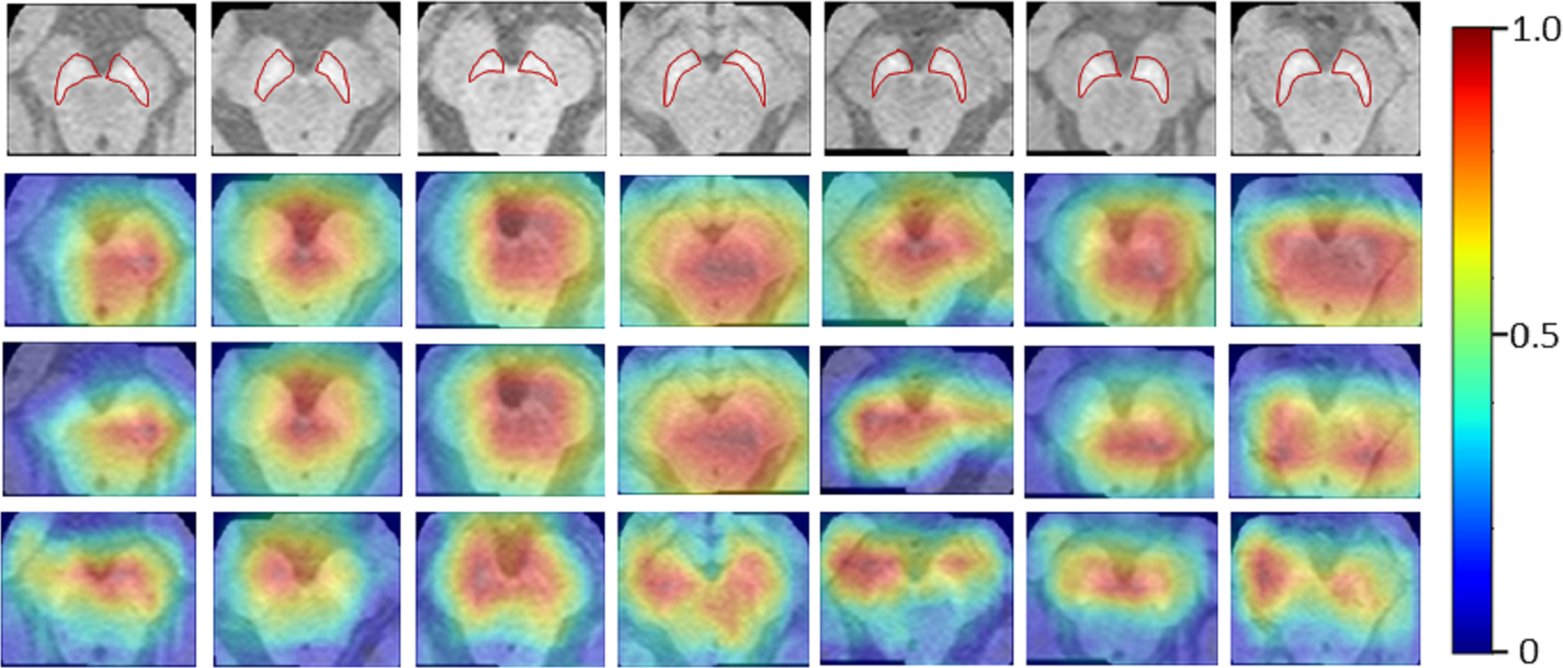
Results for the Neuromelanin MRI-PD dataset. It can be clearly observed that HR-CAMs (row 4) perform better compared to the other two CAM methods (row 2 (Zhou) and 3 (GradCAM)) resulting in activations for the left and the right SNc separately.

The Neuromelanin MRI–PD dataset comprised of 548 2D axial images (377 training and 171 testing) from 80 subjects (55-training, 25-testing). Augmentation techniques mentioned for the previous two datasets were employed to increase the size by 10 folds. The ResNet-50 base architecture was trained on the boxed regions around the brain-stem using binary cross entropy as the loss metric. The model performed with an accuracy of 76.02% on the testing set of images providing an F1-score of 0.81 and AU-ROC of 0.84. Figure 5 indicates that HR-CAMs could precisely locate the substantia-nigra pars compacta compared to the other two methods which crudely capture the substantia nigra, usually as a single blob. In multiple cases, HR-CAMs were able to capture the left and the right SNc separately whereas Zhou’s CAMs and Grad-CAMs merged the left and the right SNc into a single activation blob.

### MURA

**Figure 6:**
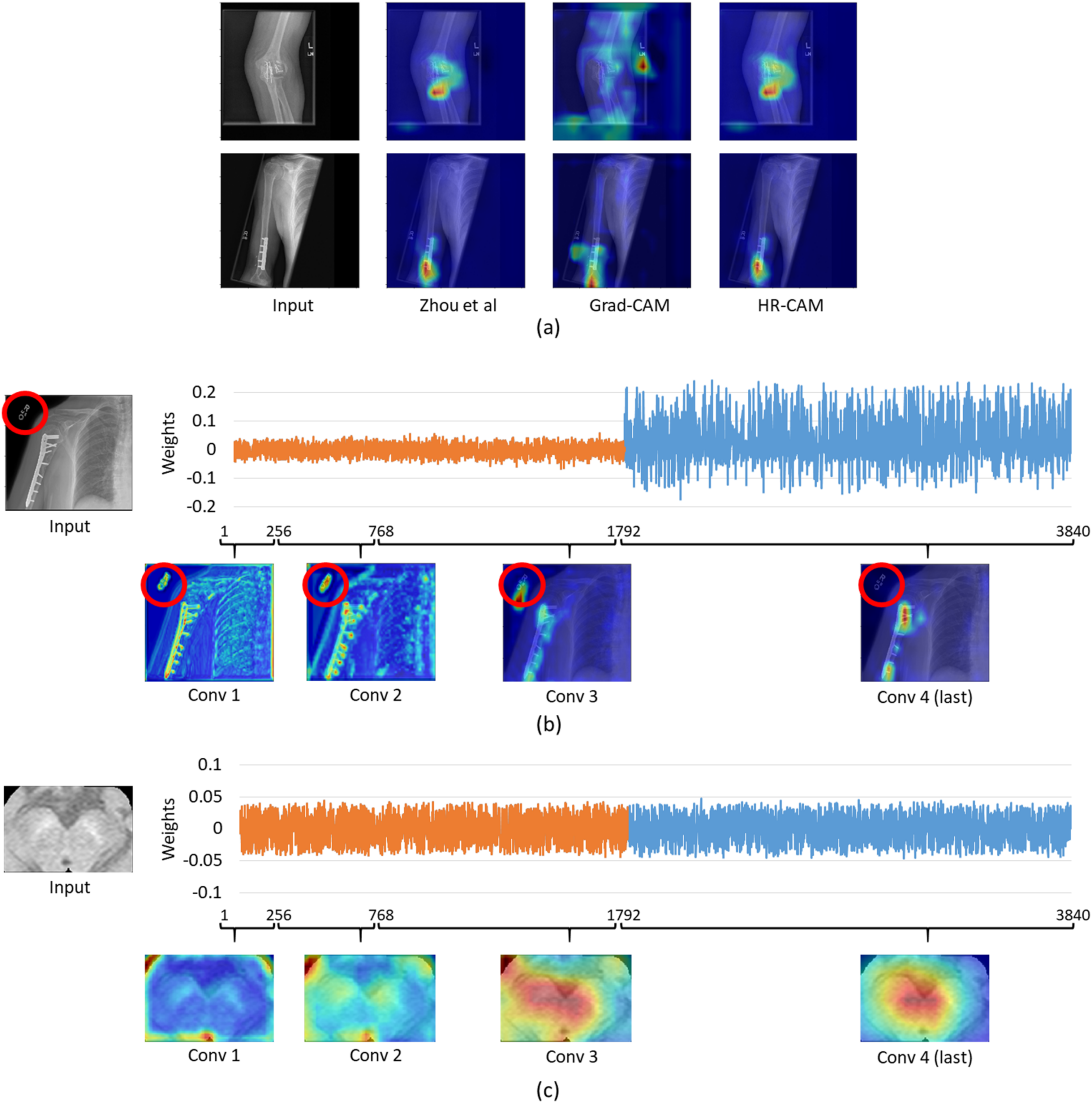
(A) Comparison of results for all the CAM techniques on the MURA dataset; (B) weights distribution of all the feature maps from the concatenated layer for an image from the MURA dataset; (C) weights distribution of all the feature maps from the concatenated layer for an image from the Neuromelanin MRI-PD dataset.

For the musculoskelatal radiographs dataset, two separate models were trained. Model 1 was the ResNet-50 architecture (Fig. 1) while model II involved the VGG-19 architecture (Simonyan & Zisserman, 2014). The models were trained on 36,803 images and tested on 3,197 images which were augmented to produce a robust classification model. Augmentation methods like flip, shift and rotate were included. The base ResNet-50 classifier provided a balanced class accuracy of 76.35% with an F1-score of 0.72 and AU-ROC of 0.83 on the testing data while the VGG-19 based model provided an accuracy of 71.91% with the F1-score of 0.70 and an AU-ROC of 0.77. Figure 6A compares all three CAM methods for the MURA dataset. In this case it was observed that the results for Zhou’s CAMs and HR-CAMs were almost identical while Grad-CAMs could not perform at par with these two methods.

To evaluate the similar performance of HR-CAM model with CAMs from Zhou et al. (2016) we mapped weights for every feature map for every sampled convolutional layer as shown in Fig. 6B. The feature maps from the initial and intermediate convolutional layers had lower weights (pertaining to localization of the letters “L” and “R” or other written information on the X-ray images) compared to the feature maps belonging to the final convolutional layer.

On the contrary, when we compared the weights from neuromelanin MRI-PD dataset (from “Neuromelanin MRI for PD”) the distribution of weights was more uniform over all the sampled convolutional layers for the HR-CAM model facilitating more accurate maps. As shown in Fig. 6C, the activation maps obtained separately for every convolutional layer show gradual increase in the focus on the SNc.

## Discussion

In this work, we proposed a generalized approach called HR-CAMs for enhanced visual explanations from CNN based architectures. We extensively validated this technique on multiple radio-pathological images to demonstrate high precision in extracting the activation maps that could be significantly valuable for clinical interpretation of pathology. The validation was not only performed on simulated datasets but also on benchmark open datasets such as ISIC and MURA where we illustrated that HR-CAMs facilitated better discriminative representations than existing methods.

Radiology images such as MRI and CT inherently have a lower resolution when compared to images captured from other sources such as digital cameras. Therefore, when a deep CNN model is applied to such radiological images, as a result of multiple max-pool layers, the image represented in the final layer is usually of poor resolution. Current CAM techniques such as from Zhou et al. (2016) extract the information obtained from such a low resolution final layer of the CNN to compute the activation maps in the same space. These maps are then upsampled to the original image size where a simple bilinear or bicubic interpolation results in the loss of finer details facilitating a blurred and imprecise visual explanation. Other techniques such as Grad-CAMs can use any layer of choice to capture the discriminative information, however is limited to information only from one layer. We mitigated these shortcomings through the HR-CAM technique where we employed input from all the pre-max-pooling layers to create an optimized class activation map.

Initial validation was performed on a simulated dataset where the baseline abnormality was known. The classifier performed with a high accuracy and the CAMs produced by our technique focused on the fine patterns of abnormality that were induced as compared to coarse maps provided by the other two techniques. To demonstrate the consistency of our superior results on all types of simulated abnormalities (in 4,000 images) quantitative inference was provided in Fig. 3. The box-plots illustrated that the median sensitivity and specificity was significantly high in HR-CAMs. Moreover, a statistical test demonstrated that HR-CAMs were highly sensitive to the abnormality and were more specific than GradCAMs.

We also applied our technique on benchmark dataset of ISIC and illustrated similar findings as the simulated data, where HR-CAMs facilitated superior maps as shown in Fig. 4. The weights from each layer included in the HR-CAM model were trained to facilitate the most discriminative map via the final GAP layer as shown in Fig. 1. On similar lines, we demonstrated the maps even on a small dataset of neuromelanin sensitive MRI to classify PD from healthy controls were more specific implying that regardless of the size and type of data HR-CAMs have the potential to facilitate precise and enhanced visual explanations than other comparative methods.

For the MURA data, we observed that the maps from HR-CAM and Zhou’s CAMs were similar while the ones given by GradCAM were inferior. To achieve a profound understanding of why HR-CAMs performed at par with CAMs were mapped all the trained weights to observe that the earlier layers were not weighted (zero weight) compared to the final layers implying that the sampled initial and intermediate convolutional layers did not focus on the intended area of interest. This was mainly due to the presence of artifacts/redundant information (in this case written information on x-rays such as “L” or “R” or other written information that varied from scan to scan) in the input data which the model learned with the initial and intermediate convolutional layers (as shown in Fig. 6). The activation maps extracted from every convolutional layer, facilitated evidence that the HR-CAM architecture learns the most discerning areas and discards the redundant features. Therefore, the feature maps from the final convolutional layer were weighed exceedingly high compared to the other layers. Since Zhou’s CAMs take into consideration only the final layer of the network, most of the useful information pertaining to the distinguishing areas was captured and hence were identical to HR-CAMs.

Limitations of HR-CAM include freezing of layers that makes the framework a two-step procedure. Nonetheless, HR-CAMs are highly adaptable to the dataset under consideration and consequently facilitate superior CAMs compared to the existing methods. Overall, the technique with its validation on multiple datasets lends credence for clinical explanation and interpretation.

## Supplemental Information

10.7717/peerj-cs.622/supp-1 Supplemental Information 1 Code for HR-CAM in Python.

## Additional Information and Declarations

### Data Availability

The following information was supplied regarding data availability: The code for tumor HR CAMs is available in the Supplemental File. The data were obtained from third-parties: 1. The ‘Neuromelanin MRI for PD’ dataset is available at NIMHANS. The authors confirm that all data underlying the findings will be made available if requested. Data access will be provided upon the approval of the Institutional Ethics Committee. Contact details are: The Member secretary, NIMHANS Ethics Committee, NIMHANS, Bangalore, INDIA; Phone No. 080-26995004 or Email: deannimhans@yahoo.com. 2. The MURA (musculoskeletal radiographs) dataset is available at Picture Archive and Communication System (PACS), Stanford Hospital: https://stanfordmlgroup.github.io/competitions/mura/. 3. ISIC data is available at the International Skin Imaging Collaboration (ISIC)’s archive. The datasets used were HAM10000 and Derm7 and they are available at https://challenge.isic-archive.com/data.
